# A cellular overview of immunometabolism in systemic lupus erythematosus

**DOI:** 10.1093/oxfimm/iqad005

**Published:** 2023-05-11

**Authors:** Antonios Psarras, Alexander Clarke

**Affiliations:** Kennedy Institute of Rheumatology, NDORMS, University of Oxford, Oxford, UK; Kennedy Institute of Rheumatology, NDORMS, University of Oxford, Oxford, UK

**Keywords:** systemic lupus erythematosus, autoimmunity, immunometabolism, T cells, B cells

## Abstract

Systemic lupus erythematosus (SLE) is a complex autoimmune disease, characterized by a breakdown of immune tolerance and the development of autoantibodies against nucleic self-antigens. Immunometabolism is a rapidly expanding scientific field investigating the metabolic programming of cells of the immune system. During the normal immune response, extensive reprogramming of cellular metabolism occurs, both to generate adenosine triphosphate and facilitate protein synthesis, and also to manage cellular stress. Major pathways upregulated include glycolysis, oxidative phosphorylation, the tricarboxylic acid cycle and the pentose phosphate pathway, among others. Metabolic reprogramming also occurs to aid resolution of inflammation. Immune cells of both patients with SLE and lupus-prone mice are characterized by metabolic abnormalities resulting in an altered functional and inflammatory state. Recent studies have described how metabolic reprogramming occurs in many cell populations in SLE, particularly CD4^+^ T cells, e.g. favouring a glycolytic profile by overactivation of the mechanistic target of rapamycin pathway. These advances have led to an increased understanding of the metabolic changes affecting the inflammatory profile of T and B cells, monocytes, dendritic cells and neutrophils, and how they contribute to autoimmunity and SLE pathogenesis. In the current review, we aim to summarize recent advances in the field of immunometabolism involved in SLE and how these could potentially lead to new therapeutic strategies in the future.

## Introduction

Systemic lupus erythematosus (SLE) is a complex autoimmune disease, characterized by a breakdown of immune tolerance and the development of autoantibodies against nucleic self-antigens. SLE usually develops in a stepwise fashion over many years. Autoantibodies appear at an early stage, when patients are still asymptomatic, many years before clinically overt disease [1]. Both innate and adaptive immune mechanisms are involved in the development of SLE, which leads to the activation of multiple cell types, inflammatory cascades, complex immunological networks and eventually end-organ tissue damage. Despite advances in determining the key immunological pathways involved in the pathogenesis of SLE, the disease remains incompletely understood.

The metabolic state of immune cells is emerging as a critical checkpoint of their effector and regulatory functions, including proliferation and activation, secretion of molecules (e.g. cytokines and chemokines), migration to tissues and escalation or control of inflammation. All these processes are highly metabolically demanding, necessitating high uptake of valuable nutrients such as glucose, amino acids and fatty acids in order to generate adenosine triphosphate (ATP). The metabolic state is dependent on two key pathways: glycolysis and oxidative phosphorylation (OXPHOS). Glycolysis involves the conversion of glucose into pyruvate, which may then enter the tricarboxylic acid (TCA) cycle and be oxidized, or be converted to lactate, which usually occurs under anaerobic conditions. Although oxidation of pyruvate in the TCA cycle yields many more ATP molecules per glucose molecule than conversion to lactate (∼36 vs 2), activated immune cells typically prefer aerobic glycolysis (i.e. production of lactate despite normoxia), which allows high flux of glycolytic intermediates which can be used for biosynthesis or redox balance [2]. OXPHOS is the metabolic pathway in which ATP is generated via oxidation of pyruvate in the mitochondria. Electrons supplied by NADH are transferred through an electron transport chain across the inner mitochondrial membrane, generating an electrochemical gradient which powers ATP synthesis. Changes in the amount and type of nutrients used following activation of the immune response, as well as the metabolic processes by which they are utilized by the cells, have been referred to as metabolic reprogramming. Immunometabolism is a rapidly expanding scientific field investigating the metabolism of immune cells. In patients with SLE as well as lupus-prone mice, metabolic abnormalities in T cells were first reported over 15 years ago [3, 4].

## Genetic and epigenetic factors

Metabolic reprogramming of immune cells has been studied in patients with SLE and lupus-prone mice, with most focus on T cells [5]. These processes are complex and are likely to be influenced by genetic and epigenetic factors. Genetic factors have been associated with mitochondrial dysfunction in autoimmunity. *Sle1c2*, a lupus susceptibility locus in mice, is associated with a decreased level of estrogen-related receptor gamma, a mitochondrial metabolism regulator and mitochondrial dysfunction [6]. The *UCP2* (uncoupling protein 2) –866 G/A polymorphism, a gene involved in both mitochondrial ATP production and reactive oxygen species (ROS) generation, has been associated with SLE and rheumatoid arthritis [7]. In contrast, the AG and AA genotypes were associated with decreased risks of both diseases when compared with GG genotype [8, 9].

Mechanistic target of rapamycin (mTOR) is a sensor system which can form two complexes, mTOR complex 1 (mTORC1) and complex 2 (mTORC2). mTOR is crucial for the integration of metabolic signals regulating cellular growth, homeostasis and energy use. mTOR functions as a serine/threonine protein kinase and its signalling pathway is regulated by metabolic cues (e.g. glucose and amino acids) as well as by growth factors, hormones and cytokines. mTOR appears to be involved as a major regulator in various rheumatic diseases [10]. Signal transduction via the Rag family of small GTPases mediates the translocation of mTORC1 from the cytoplasm to the surface of the lysosome, where mTORC1 is activated by GTP-binding protein Rheb, in response to amino acid availability [11]. Genetic activation of the mTORC1 pathway was also reported to be associated with SLE, as lupus-like pathology was observed in patients suffering from tuberous sclerosis, in which mutations in the genes encoding hamartin (TSC1) or tuberin (TSC2) form the TSC complex which functions as inhibitor of mTORC1 activation [12–14]. This leads to unrestrained activation of mTORC1 signalling.

Epigenetic processes are crucial in SLE and can regulate gene expression via DNA methylation, post-translational histone modifications and microRNAs [15]. DNA methyltransferases are impaired in SLE T cells as a result of mitochondrial dysfunction [16]. Profiling of the levels of metabolites in sera from SLE patients revealed profound lipid peroxidation, reflective of oxidative damage, suggesting that additional defects in the *S*-adenosyl-l-methionine cycle might contribute to DNA hypomethylation. Apart from DNA methylation, acetylation of histone and non-histone proteins is key mediators in SLE. Histone deacetylases are overexpressed in T cells from MRL/*lpr* lupus-prone mice, which could be secondary to protein nitration and oxidative stress given that histone deacetylation is NAD^+^-dependent [17].

## Metabolism of T cells

T cells are key players in initiation and perpetuation of autoimmunity in SLE, characterized by well-established alterations in signalling, cytokine production, proliferation and other regulatory functions [18]. CD4^+^ T cells in SLE display an altered signalling phenotype, with rewiring of their T-cell receptor (TCR) signalling. Decreased expression of the CD3ζ chain and replacement by the homologous Fcγ receptor chain result in downstream signalling through Syk kinase instead of the normal CD3ζ partner Zap70 [19]. The lysosomal degradation of the CD3ζ chain is a consequence of increased oxidative stress in lupus T cells [1].

Signalling via mTORC1 is essential for the polarization of naïve T cells to type 1 T helper (T_H_1) and type 17 T helper (T_H_17) cells in both healthy donors and patients with autoimmune rheumatic diseases [10, 20]. In regulatory T cells (T_reg_), inhibition of mTORC1 was reported to promote their expansion, but other data suggested that mTORC1 is an essential requirement for T_reg_ suppressive function [10, 21]. Although CD4^+^ follicular helper T (T_FH_) cells do not activate the mTORC1 pathway upon viral infection, inhibition of AMP-activated protein kinase (AMPK) and subsequent activation of mTORC1 induce T_FH_ cell differentiation and lupus-like disease in mouse models [22–24]. A CXCR5^−^CXCR3^+^PD1^hi^CD4^+^ helper T-cell population distinct from T_FH_ cells was also reported to expand in peripheral blood and the renal tubulointerstitial areas of patients with proliferative lupus nephritis [25]. These cells were found to accumulate mitochondrial ROS because of reverse electron transport fuelled by succinate, actively promoting B-cell activation through supply of IL-10 and succinate.

Many mechanisms have been reported to result in mTORC1 activation in SLE T cells, such as mitochondrial dysfunction, pentose phosphate pathway (PPP) activation, high activity of transaldolase and accumulation of kynurenine, a tryptophan metabolite with immune modulatory functions [26, 27]. Iron metabolism was also shown to play an active role in T-cell function. Activated T cells upregulated both transferrin receptor (CD71) and iron uptake via increased endosomal recycling, features which were exaggerated in lupus T cells [28]. Blockage of transferrin receptor led to reduction of intracellular iron and mTORC1 signalling, which in turn inhibited T_H_1 and T_H_17 cells but enhanced T_reg_ differentiation and was associated with amelioration of disease severity in lupus-prone mice.

Glycolysis has an important role in the effector functions and cytokine production of T cells. CD4^+^ T cells from patients with SLE and lupus-prone mice are characterized by enhanced glycolysis [29, 30]. T-cell activation via TCR and CD28 stimulation induces GLUT1 expression, which correlates with increased glucose uptake and glycolysis [31]. Although overexpression of GLUT1 in mice was associated with cell activation and production of autoantibodies, this was not a universal feature of T cells in SLE patients [32–34]. A study investigating the correlation between GLUT1 expression and SLE disease activity found no significant difference in gene expression of *GLUT1* among healthy controls, SLE with low and high disease activity. However, surface expression of GLUT1 on effector memory CD4^+^ T cells measured by flow cytometry was higher in SLE patients with high disease activity (SLEDAI ≥8) than in healthy controls or SLE patients with low disease activity (SLEDAI <8) [35].

**Figure 1.**
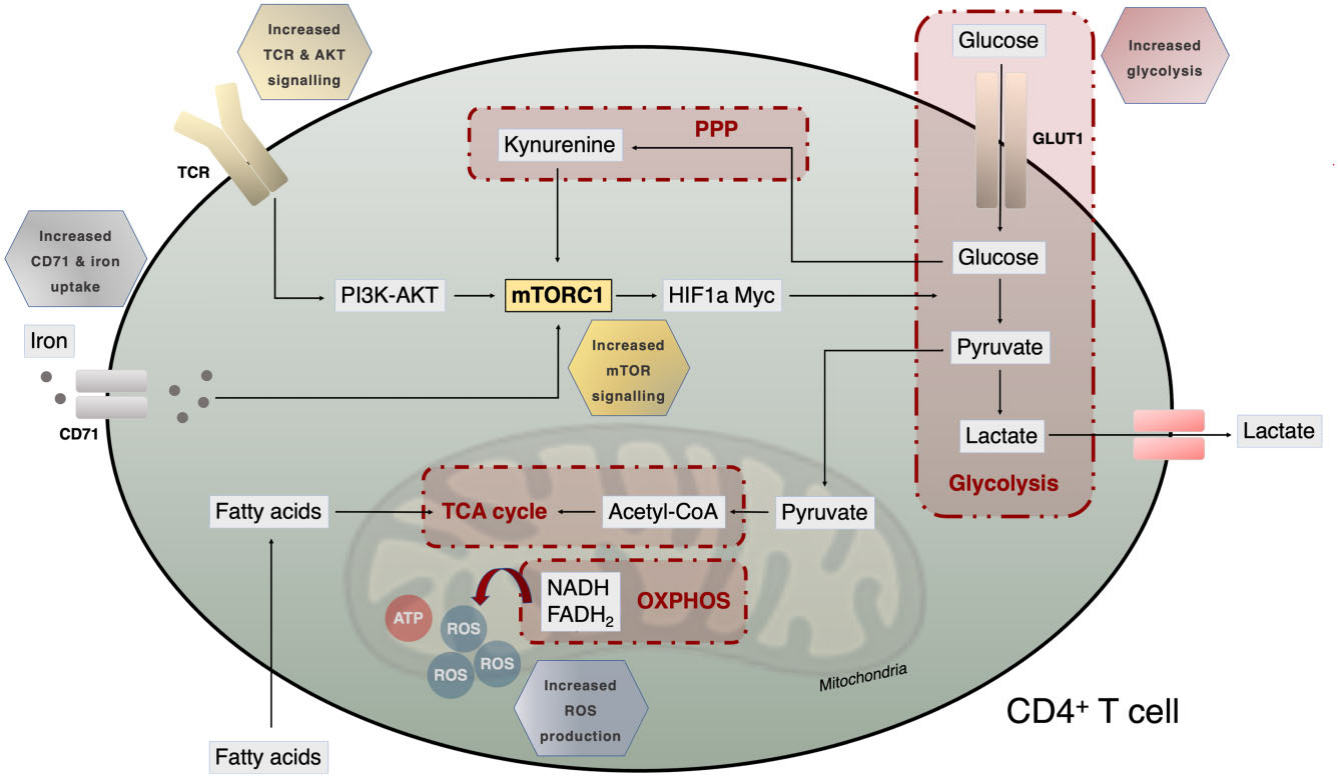
Metabolic reprogramming of CD4^+^ T cells in SLE. CD4^+^ T cells from patients with SLE and lupus-prone mice are characterized by enhanced glycolysis, in which glucose is converted into pyruvate. Pyruvate enters the TCA cycle to generate NADH and FADH_2_ or it is further metabolized into lactate, which is secreted by the cell. NADH and FADH_2_ will in turn enter the electron transport chain (ETC) to generate ATP via OXPHOS. Glucose can also be metabolized via the PPP, which leads to accumulation of kynurenine and activation of mechanistic target of rapamycin complex 1 (mTORC1). TCR stimulation can also activate mTORC1 through the PI3K–AKT pathway. mTORC1 activation via TCR activation can induce glucose metabolism through HIF-1α and Myc proto-oncogene protein, which itself contributes to mitochondrial dysfunction. CD4^+^ T cells can utilize fatty acids as a source of energy by degrading fatty acids through fatty acid oxidation. Activated T cells also upregulate transferrin receptor (CD71) and iron uptake via increased endosomal recycling, which in turn promotes differentiation and cell activation.

Glucose deprivation leads to decreased intracellular ATP levels and activation of the serine/threonine kinase AMPK, which has a positive regulatory effect on signalling pathways compensating for the lack of cellular ATP [36]. The phosphatidylinositol 3-kinase (PI3K)-AKT signalling pathway is a crucial signal transduction pathway regulating cellular survival, growth, proliferation and migration, in which PI3K and AKT (protein kinase B) have important roles [37]. PI3K activation phosphorylates and activates AKT, which is in turn translocated to the plasma membrane; this pathway eventually leads to activation of mTORC1. T-cell activation through AKT signalling following stimulation supports both increased glycolysis and OXPHOS [38, 39]. The enhanced glycolysis and OXPHOS found in naive CD4^+^ T cells from lupus-prone mice correlated with cellular activation status, especially excessive interferon gamma (IFNγ) production [29]. Normalization of T-cell metabolism *in vitro* through dual inhibition of glycolysis and mitochondrial metabolism could be a therapeutic avenue for SLE. Interestingly, this high glycolytic function and mitochondrial respiration observed in SLE T cells was also observed in effector memory CD4^+^ T cells from healthy controls, and their population is expanded in patients with SLE [40, 41]. Reduction in glycolysis via inhibition of glutaminase 1 was reported to ameliorate disease in MRL/*lpr* lupus-prone mice in a T_H_17-dependent manner [42]. In lupus-prone mice, the hypoxic environment associated with renal tissue injury was shown to upregulate hypoxia-inducible factor-1 (HIF-1) in CD4^+^ and CD8^+^ T cells, resulting in metabolic reprogramming and subsequently in increased effector function and resistance to apoptosis [43]. An overview of CD4^+^ T-cell metabolism and how this is affected in SLE can be seen in Fig. 1.

Early and significant dysfunction of the glycosphingolipid metabolic pathway in the kidneys of lupus-prone mice and patients with lupus nephritis has been reported, indicating the importance of lipid metabolism in the pathogenesis of the disease [44]. Cholesterol and glycosphingolipids are important components of lipid rafts of the cell plasma membrane and are aggregated in T cell from SLE patients [45]. Inhibition of glycosphingolipid biosynthesis *in vitro* was shown to normalize glycosphingolipid metabolism, to correct CD4^+^ T-cell signalling and functional abnormalities and to decrease anti-dsDNA antibody production by autologous B cells in SLE patients [46]. Friend leukaemia integration 1 (FLI1) is a transcription factor targeting neuraminidase 1, which is involved in glycosphingolipid synthesis. *FLI1* haplodeficiency in MRL/*lpr* lupus-prone mice was shown to decrease the pathogenicity of T cells by reducing TCR-specific activation and IL-4 production in part through the modulation of glycosphingolipid metabolism [47]. Interestingly, a variant in the *FLI1* promoter region resulting in increased *FLI1* expression was associated with susceptibility to SLE [48].

Cholesterol is a central regulator of TCR signalling and effector functions in CD8^+^ T cells [49]. Inhibiting cholesterol esterification in T cells by genetic ablation or pharmacological inhibition of acetyl-CoA acetyltransferase 1, a key cholesterol esterification enzyme, resulted in enhanced proliferation and activation of CD8^+^ but not CD4^+^ T cells, via enhanced T-cell receptor clustering and signalling in the immunological synapse. How lipid metabolism is affected in CD8^+^ T cells of SLE patients is not yet established.

The IFN system is activated in the majority of SLE patients and is associated with profound immunological abnormalities [50, 51]. A study investigating how type I IFNs affect the metabolic state of CD8^+^ T cells in SLE patients reported that the downregulation of mitochondria-derived genes and mitochondria-associated metabolic pathways was associated with a high type I IFN signature in lupus patients [52]. Additionally, CD8^+^ T cells from these patients had enlarged mitochondria and lower spare respiratory capacity associated with increased cell death upon rechallenge with TCR stimulation. Upon stimulation with type I IFNs and TCR ligation of CD8^+^ T cells isolated from healthy controls, mitochondrial abnormalities could be reproduced in a similar manner seen in SLE patients. These data signify that type I IFNs can induce metabolic rewiring of CD8^+^ T cells by increasing NAD^+^ consumption, which in turn promotes impaired mitochondrial respiration and cell death. Interestingly, mutations in *ATAD3A*, which encodes ATPase family AAA domain-containing protein 3A, were reported to upregulate interferon-stimulated genes in patients with mitochondrial disease, a process mediated by enhanced activation of cyclic GMP-AMP synthase (cGAS) and stimulator of interferon genes (STING) [53].

A large-scale bulk RNA-sequencing study of 27 immune cell types in 136 SLE patients identified distinct transcriptomic signatures associated with clinical features such as organ involvement and response to therapy [54]. Interestingly, different enrichment patterns were reported between the signatures of metabolism-related and cellular-related pathways. For instance, TCA cycle genes showed enrichment in activity signatures in memory CD8-lineage cells. On the other hand, ribosome and cell cycle pathways were enriched predominantly in disease-activity signatures of T_H_1 cells and memory CD8-lineage cells but also in non-T-cell subsets such as NK cells. The cell-type-specific analysis successfully clarified the cell-type origin of these pathways and highlighted the importance of immunometabolism in disease establishment and exacerbation phases.

## Metabolism of B cells

B cells contribute to lupus pathogenesis via antigen presentation, autoantibody production, cytokine production and interaction with other immune cells. Autophagy is a critical homeostatic mechanism for plasmablast development, which was previously shown to play a central role in early developmental and transitional stages of autoreactive B cells in a lupus model via the induction of significant cellular stress [55].

Similarly to T cells, activated B cells predominantly acquire a glycolytic profile [56]. In lupus-prone mice, mTORC1 is overexpressed in B cells, enhancing plasma cell differentiation and autoantibody production, while mTOR pathway inhibition by rapamycin decreased B-cell proliferation and survival [57, 58]. Interestingly, high levels of B-cell-activating factor (BAFF), an important mediator of B-cell survival and therapeutic target in SLE patients, were found to increase glucose metabolism and glycolysis via mTORC1 activation and promotion of protein synthesis [59, 60]. Induction of glycolysis was shown to be critical for antibody production by plasma cells in BAFF transgenic mice, while inhibition of glycolysis with the pyruvate dehydrogenase kinase inhibitor dichloroacetate significantly suppressed B-cell proliferation and antibody secretion both *in vitro* and *in vivo* [59]. Long-lived plasma cells are central in the pathogenesis of SLE, producing autoantibodies (e.g. anti-dsDNA, anti-Ro, anti-La, anti-Sm, anti-RNP and anti-cardiolipin) [61]. In NZB/W F1 lupus-prone mice, autoreactive long-lived plasma cells were shown to be regenerated within 2 weeks after depletion by using the proteasome inhibitor bortezomib [62]. The differentiation of B cell into a long-lived plasma cell is largely dependent on both intrinsic (e.g. BTB transcription factor ZBTB20) and extrinsic factors (e.g. APRIL, BAFF, IL-6) [63–65]. Interestingly, survival of long-lived plasma cells was also shown to require mitochondrial pyruvate import via the mitochondrial pyruvate carrier (MCP) complex [66]. Long-lived plasma cells demonstrated higher glucose uptake in comparison with short-lived plasma cells and this glucose was essential for the generation of pyruvate. Glucose was primarily used to glycosylate antibodies, but deletion of *Mpc2*, an essential component of the MCP, led to a progressive loss of long-lived plasma cells and of vaccine-specific antibodies *in vivo*. The above results may indicate that inhibition of glucose utilization could target long-lived plasma cells by preventing antibody glycosylation as well as by impairing cell survival through lack of pyruvate.

Fatty acid amide hydrolase (FAAH) can degrade ligands for cannabinoid receptors and members of the peroxisome proliferator-activated receptor (PPAR) family and is encoded by the metabolic gene *Faah*. *Faah* has been proposed as a susceptibility gene in the murine NZM2410-derived *Sle2* locus [67]. Although increased *Faah* expression associated with the *Sle2* locus did not breach central immune tolerance in a transgenic B-cell receptor model, it promoted B-cell receptor revision in mature B cells via high expression of endogenous Ig H and L chains in splenic B cells and upregulation of recombination activating genes (RAG), resulting in enhanced autoantibody production. Increased levels of FAAH were also reported in plasma cells from patients with SLE [68]. Although the underlying mechanism connecting metabolism of fatty acid amides and esters to B-cell function remains unclear, PPARγ agonists in lupus-prone mice showed a beneficial effect ameliorating disease activity, atherosclerosis, hypertension and overall organ damage [69–72].

OXPHOS genes were found to be enriched in disease-state signatures in B-lineage cells from SLE patients in a recent large-scale transcriptomic study [54]. Another study investigating metabolic changes in B cells from SLE patients demonstrated that staining with DiOc6 (indicating mitochondrial membrane polarization) was higher in B cells from SLE patients than in healthy controls, and was positively correlated to the percentage of plasmablasts in the peripheral blood as well as disease-activity scores [73]. TLR9 and IFNα stimulation enhanced glycolysis, OXPHOS and DiOc6 staining in B cells, further promoting plasmablast differentiation *in vitro*. Importantly, in the absence of glutamine, both glycolysis and OXPHOS were reduced, suppressing plasmablast differentiation.

## Metabolism of monocytes and macrophages

Metabolic reprogramming of monocytes and macrophages during inflammatory responses is less studied in SLE compared with T-cell subsets. Monocytes exhibit polyfunctional cytokine expression patterns, while newly diagnosed untreated SLE patients share a distinct monocytic chemokine signature despite their clinical heterogeneity [74]. Reduced numbers of tingible body macrophages, along with impaired ability to efficiently clear apoptotic cellular debris have been described in the germinal centres of SLE patients [75]. A link between the aberrant type I IFN production seen in SLE and monocytic function was established, as IFNα can directly impair the autophagy-mediated degradation of mitochondrial DNA (mtDNA). This defect results in promotion of autoreactivity of SLE monocytes in a STING-dependent fashion [76]. Intracellular sensing of mtDNA via the cGAS-STING pathway can induce a type I IFN response, a process that typically characterized by impaired OXPHOS and ATP production, loss of mitochondrial potential and mROS induction, leading to a loss of mitochondrial integrity and release of mitochondrial components [77]. Of note, inhibition of mTOR signalling pathway *in vitro* with rapamycin was shown to reduce type I IFN production by SLE monocytes [78]. Inhibition of fumarate hydratase was shown to increase IFNβ production in macrophages through mechanisms driven by mtRNA release and activation of the RNA sensors TLR7, RIG-I and MDA5 [79]. Macrophages from SLE patients were demonstrated to exhibit suppressed levels of fumarate hydratase, suggesting a pathogenic mechanism in sustaining type I IFN responses.

Macrophages utilize arginine as an important energy source in two main metabolic pathways: the nitric oxide synthesis pathway through classical activation and the arginase pathway through alternative activation [80]. The nitric oxide synthesis pathway was linked to an inflammatory M1 phenotype with nitric oxide synthase being the main mediator [81]. M1 macrophages typically exhibit a glycolytic phenotype. On the other hand, for M2 macrophages, the production of α-ketoglutarate via glutaminolysis is essential for their polarization, while fatty acid oxidation and mitochondrial respiration are the primary pathways for their functional requirements [82]. Glutamine catabolism is an important regulator, as it is essential for IL-1 induction by macrophages upon lipopolysaccharide (LPS) stimulation [83, 84]. Glutamine can also be incorporated in the TCA cycle and the hexosamine pathway; this can induce M2 macrophage polarization upon IL-4 stimulation. However, glutamine is not a requisite for the development of LPS-stimulated M1 macrophages [85]. Overall, M2 macrophages are characterized by higher basal mitochondrial oxygen consumption rates [86, 87]. Despite the better understanding of the above pathways and their role in macrophage biology, the precise metabolic rewiring in different macrophage subsets in SLE remains unclear.

Macrophages are also capable of taking up various types of lipids such as low-density lipoprotein, very-low-density lipoprotein and oxidized lipoproteins through processes including phagocytosis, macropinocytosis and scavenger receptor-mediated pathways [88]. Macrophages from SLE patients are known to have impaired phagocytic capacity. Fatty acid oxidation was shown to regulate multiple inflammatory functions of macrophages as well as macrophage differentiation [89]. Abnormal deposition of fatty acids and lipoproteins in macrophages can contribute to foam cell formation and induction of inflammation, with particular emphasis on unsaturated fatty acids (e.g. oleic acid, linoleic acid and arachidonic acid), which were shown to induce IL-1α secretion by foam cells *in vivo* [90, 91].

Splenic marginal zone macrophages are an important population actively contributing to the tolerogenic clearance of apoptotic cells and debris via indoleamine-2,3-dioxygenase (IDO) [92]. Intracellular signalling via IDO can induce the metabolic-stress sensing kinase general control non-derepressible 2 (GCN2), which phosphorylates eIF-2α, the activity of which can prevent autoimmune phenomena mediated by an excessive amount of apoptotic material [93]. In response to tryptophan catabolism mediated by IDO, GCN2 can induce a stress response that regulates innate immunity [94].

## Metabolism of neutrophils

Neutrophils are dysfunctional in SLE patients, with impaired phagocytosis and reduced production of ROS, features associated with disease severity and end-organ damage [95, 96]. Neutrophils (particularly the subset of low-density granulocytes) also undergo a particular form of cell death named ‘NETosis’ by releasing neutrophil extracellular traps (NETs) and DNA which are immunogenic. Apart from the enhanced NETosis, SLE patients also have impaired removal of those NETs [97, 98].

Ribonucleoprotein-containing immune complexes can induce mitochondrial membrane hyperpolarization and ROS generation by blocking transcription factor A, mitochondrial (TFAM) phosphorylation, resulting in NET formation as well as oxidation of mtDNA [99]. The accumulation of oxidized mtDNA within the mitochondria of neutrophils in SLE is highly proinflammatory when extruded in NETs, inducing a strong type I IFN response. Of note, mitochondrial ROS inhibition *in vivo* reduced disease severity and attenuated type I IFN responses in a lupus mouse model, while decreased spontaneous NETosis and reduced disease activity were reported in MRL/*lpr* mice treated with a mitochondrial-ROS scavenger [100]. Interestingly, there is a feed-forward loop between NETs and macrophages in SLE patients, in which both NETs and LL-37 induce IL-18 and IL-1β secretion via activation of the inflammasome [101]. These cytokines can in turn stimulate neutrophils to undergo further NETosis, leading to an amplification of this inflammatory loop.

NADPH oxidation was also reported to have a great impact on neutrophil function by regulating NETosis [102]. NADPH oxidase 2 (Nox2) is a vital subunit of the NADPH oxidase enzymatic complex, which plays an essential role in ROS generation by phagocytes. Nox2-deficient male mice were unable to undergo NETosis, but in contrast to the hypothesis that disease will be ameliorated, the mice developed markedly exacerbated lupus with increased spleen weight, increased renal disease and elevated and altered autoantibody profiles. Intriguingly, heterozygous female mice, which have Nox2-deficiency in 50% of neutrophils on average due to X-chromosome inactivation, also developed exacerbated lupus and altered autoantibody patterns, suggesting that failure to undergo normal Nox2-dependent cell death may result in release of immunogenic self-constituents that stimulate lupus [102]. Additionally, ATP production and autocrine purinergic signalling via P2Y2 receptors might be essential for neutrophil chemotaxis via promotion of mTOR signaling [103]. Blocking mTOR signalling with rapamycin reduced mitochondrial Ca^2+^ uptake and membrane potential, and further impaired cellular ATP release and neutrophil chemotaxis. Overall, the precise metabolic rewiring necessary for the recruitment and function of neutrophils in the inflammatory response in SLE remains unclear, and it can vary among patients and across different target organs.

## Metabolism of myeloid dendritic cells

Activation of professional antigen-presenting cells such as myeloid dendritic cells (mDCs) is a crucial link between innate and adaptive immune responses. mDCs perform prolonged self-antigen presentation and pro-inflammatory cytokine production in autoimmunity, and have defective tolerogenic functions [104]. Resting mDCs are characterized by a catabolic metabolic state, continuously breaking down fatty acids and glutamine. This is mediated by OXPHOS and the TCA cycle, largely regulated by AMPK [105, 106].

mDCs also utilize intracellular glycogen to support basal glycolytic needs, which provides metabolic substrates for mitochondrial respiration [107]. Upon immunogenic activation, DCs acquire an anabolic metabolic state. Activated DCs switch to aerobic glycolysis, which increases availability of glycolytic intermediates to enter the PPP [108]. Antigen processing and presentation by mDCs requires glycolysis and glycogen metabolism along with fatty-acid synthesis to further stimulate T cell for activation and differentiation [109]. Stimulation with toll-like receptor (TLR) agonists leads to a rapid increase in glycolysis, leading to the *de novo* synthesis of fatty acids for the expansion of the endoplasmic reticulum and Golgi required for the production and secretion of proteins crucial to mDC activation. Of note, this TLR-mediated glycolytic flux is signalled via the kinases TBK1, IKKε and Akt by promoting the association of the glycolytic enzyme HK-II with mitochondria.

The mTOR signalling pathway has a crucial role in integrating signalling from TLRs and growth factors with intracellular nutrient levels [110]. Constitutive mTORC1 activation was shown to impair mDC survival and proliferation but accelerated their maturation through Myc-dependent metabolic reprogramming. This metabolic switch is characterized by high levels of ROS production [111]. Tolerogenic dendritic cells were also reported to require fatty-acid oxidation to perform their suppressive role and control inflammatory responses [112].

## Metabolism of plasmacytoid dendritic cells

Plasmacytoid dendritic cells (pDCs) are considered the professional type I IFN-producing cells during acute viral infection and they have pleiotropic effects on the immune system including both cytokine secretion and antigen presentation [51, 113]. Type I IFN production by TLR9-activated pDCs leads to significant metabolic reprogramming by promoting fatty acid oxidation and OXPHOS via an autocrine type I IFN receptor-dependent pathway [114]. These metabolic changes are necessary for pDC activation, while glucose flux and mitochondrial pyruvate uptake to the TCA cycle are required to generate citrate for *de novo* fatty acid synthesis. However, pDC function was demonstrated to be severely impaired in patients with SLE and other autoimmune conditions such as primary Sjögren’s syndrome, including TLR-dependent type I IFN production, antigen presentation and T-cell activation [115]. This impaired functional state of pDCs in SLE patients was associated with activation of intracellular pathways involved in cellular senescence and stress (*ATG14*, *ATP7A*, *DNAJB1*), protein degradation in lysosomes (*CTSL*) and negative regulation of TLR signalling (*IRAK3*). It is still unclear whether altered metabolic pathways contribute to this defective phenotype. The aberrant type I IFN activation originated from non-haematopoietic tissue cells, while this defective phenotype also extended to preclinical phases of SLE [50, 115].

## Treatment approaches

Metabolic reprogramming can affect T-cell fate and many current therapeutic agents can directly influence the immune phenotype of lymphocytes by altering the cellular metabolic state. Methotrexate is the commonest disease-modifying antirheumatic drug (DMARD) used in most inflammatory arthritides, as well as in connective tissue diseases such as SLE. Methotrexate is known to inhibit purine and pyrimidine (DNA) synthesis by activating the AMPK pathway, which in turn inhibits mTOR activation and glycolysis [116–118]. Mycophenolate mofetil (MMF), which is widely used in the treatment of lupus, inhibits inosine monophosphate dehydrogenase type II, directly impacting guanosine (DNA) synthesis.

MMF has a major effect on B-cell function, as it can inhibit both proliferation and differentiation of primary human B cells, particularly during early activation events and arrested cells in the G0/G1 phase of the cell cycle [119]. MMF may also lead to inhibition of CD4^+^ T-cell proliferation and promotion of apoptosis via reduction of AKT–mTOR pathway activation, glycolysis and oxygen consumption [120]. Many immunosuppressive drugs including mTOR inhibitors (rapamycin), calcineurin inhibitors (tacrolimus, cyclosporine A) and inhibitors of *de novo* purine synthesis (6-mercaptopurine, mycophenolic acid and methotrexate) provide examples into how modulating these metabolic checkpoints can regulate T-cell activation, differentiation and immunogenic function [116]. Pioglitazone, a selective PPARγ agonist, can inhibit the mTOR pathway and ameliorate disease activity in lupus-prone mice, and affect T-cell function in patients with SLE [72, 121].

Targeting the mTORC1 pathway with rapamycin and its analogues might provide a window to explore new therapeutic approaches targeting the metabolic status of the immune system [122]. *N*-acetylcysteine can inhibit mTORC1. A randomized, double-blind, placebo-controlled study of 36 patients with SLE demonstrated that *N*-acetylcysteine improved disease activity and was associated with increased intrinsic mitochondrial membrane potential and reduced mTOR activity in T cells [123]. In paediatric SLE with nephritis, mTOR inhibition by rapamycin reduced STAT3 activation in effector T cells and the migration of IL-17-producing T cells in inflamed kidneys [124]. Calcium/calmodulin-dependent protein kinase IV (CaMK4), which is required during T_H_17 cell differentiation, is increased in SLE. Silencing CaMK4 in T cells from patients with SLE and healthy individuals was shown to inhibit T_H_17 differentiation through reduction of IL-17A and IL-17F mRNA [125]. Treatment of MRL/*lpr* lupus-prone mice with a CaMK4 inhibitor resulted in decreased end-organ tissue damage by reduction of inflammatory cell infiltrates and a reciprocal increase in T_reg_ activity [126]. In a single-arm, open label, phase 1/2 trial of sirolimus (rapamycin) in SLE patients, there was a reduction in both SLEDAI and BILAG disease-activity scores after 12 months in 55% of patients who completed treatment [127]. Additionally, sirolimus expanded T_reg_ and CD8^+^ memory T cells and inhibited IL-4 and IL-17 production by CD4^+^ and CD4^−^CD8^−^ double-negative T cells after 12 months. Altogether, mTOR overactivation is a crucial pathway in SLE pathogenesis and its inhibition might lead to new strategies to treat this complex autoimmune condition.

## Conclusions

Recent studies have demonstrated the importance of immune metabolic reprogramming in both preclinical autoimmunity and in patients with SLE. The focus of most studies was the metabolic alterations in various T-cell subsets, which emphasized that enhanced glycolysis and overactivation of the mTOR pathway were linked to an inflammatory profile and enhanced autoreactivity. It is important to make clear that each immune cell subset exhibits unique metabolic pathways, essential for activation, proliferation and functional performance during the immune response. As type I IFNs are major drivers of SLE pathogenesis, it would be intriguing to understand if targeting that pathway by IFNAR blockade or JAK inhibition could potentially influence the metabolic reprogramming of haematopoietic and other immune cells. Future research is required to decipher the metabolic rewiring of the cells during different phases of autoimmunity, different stages of disease activity and importantly, how this is linked to response or failure of treatment. This can potentiate the development of novel therapeutic approaches in SLE, which can be cell specific, sparing the side effects of the widely used corticosteroids and other immunosuppressants.

## Conflict of interest statement

None declared.
